# Epinephrine before defibrillation in patients with shockable in-hospital cardiac arrest: propensity matched analysis

**DOI:** 10.1136/bmj-2021-066534

**Published:** 2021-11-10

**Authors:** Erin Evans, Morgan B Swanson, Nicholas Mohr, Nassar Boulos, Mary Vaughan-Sarrazin, Paul S Chan, Saket Girotra, American Heart Association’s Get With The Guidelines-Resuscitation investigators, Anne Grossestreuer, Ari Moskowitz, Dana Edelson, Joseph Ornato, Mary Ann Peberdy, Matthew Churpek, Michael Kurz, Monique Anderson Starks, Sarah Perman, Zachary Goldberger

**Affiliations:** 1 Department of Emergency Medicine, Department of Internal Medicine, University of Iowa Carver College of Medicine, Iowa City, IA, USA; 2 Department of Epidemiology, University of Iowa College of Public Health, Iowa City, IA, USA; 3 Division of Pulmonary and Critical Care, Department of Internal Medicine, University of Iowa Carver College of Medicine, Iowa City, IA, USA; 4 Division of General Internal Medicine, Department of Internal Medicine, University of Iowa Carver College of Medicine, Iowa City, IA, USA; 5 Centre for Access Delivery Research and Evaluation, Iowa City Veterans Affairs Medical Centre, Iowa City, IA, USA; 6 Saint Luke’s Mid America Heart and Vascular Institute, University of Missouri-Kansas, Kansas City, MO, USA; 7 Division of Cardiovascular Medicine, Department of Internal Medicine, University of Iowa Carver College of Medicine, Iowa City, IA, USA

## Abstract

**Objective:**

To determine the use of epinephrine (adrenaline) before defibrillation for treatment of in-hospital cardiac arrest due to a ventricular arrhythmia and examine its association with patient survival.

**Design:**

Propensity matched analysis.

**Setting:**

2000-18 data from 497 hospitals participating in the American Heart Association’s Get With The Guidelines-Resuscitation registry.

**Participants:**

Adults aged 18 and older with an index in-hospital cardiac arrest due to an initial shockable rhythm treated with defibrillation.

**Interventions:**

Administration of epinephrine before first defibrillation.

**Main outcome measures:**

Survival to discharge; favorable neurological survival, defined as survival to discharge with none, mild, or moderate neurological disability measured using cerebral performance category scores; and survival after acute resuscitation (that is, return of spontaneous circulation for >20 minutes). A time dependent, propensity matched analysis was performed to adjust for confounding due to indication and evaluate the independent association of epinephrine before defibrillation with study outcomes.

**Results:**

Among 34 820 patients with an initial shockable rhythm, 7054 (20.3%) were treated with epinephrine before defibrillation, contrary to current guidelines. In comparison with participants treated with defibrillation first, participants receiving epinephrine first were less likely to have a history of myocardial infarction or heart failure, but more likely to have renal failure, sepsis, respiratory insufficiency, and receive mechanical ventilation before in-hospital cardiac arrest (standardized differences >10% for all). Treatment with epinephrine before defibrillation was strongly associated with delayed defibrillation (median 4 minutes *v* 0 minutes). In propensity matched analysis (6569 matched pairs), epinephrine before defibrillation was associated with lower odds of survival to discharge (22.4% *v* 29.7%; adjusted odds ratio 0.69; 95% confidence interval 0.64 to 0.74; P<0.001), favorable neurological survival (15.8% *v* 21.6%; 0.68; 0.61 to 0.76; P<0.001) and survival after acute resuscitation (61.7% *v* 69.5%; 0.73; 0.67 to 0.79; P<0.001). The above findings were consistent in a range of sensitivity analyses, including matching according to defibrillation time.

**Conclusions:**

Contrary to current guidelines that prioritize immediate defibrillation for in-hospital cardiac arrest due to a shockable rhythm, one in five patients are treated with epinephrine before defibrillation. Use of epinephrine before defibrillation was associated with worse survival outcomes.

## Introduction

Nearly 300 000 patients have an in-hospital cardiac arrest in the United States each year,^1^ and about 25% survive to discharge.^2^ In addition to prompt cardiopulmonary resuscitation, the immediate treatment of in-hospital cardiac arrest is dictated by the cardiac arrest rhythm.^3^ In patients with a non-shockable rhythm of asystole or pulseless electrical activity, guidelines including adult cardiac life support protocols recommend epinephrine (adrenaline) every three to five minutes.^4^ In contrast, for patients with a shockable rhythm of ventricular fibrillation or pulseless ventricular tachycardia, prompt defibrillation (within two minutes) is recommended, with epinephrine reserved for patients in whom ventricular fibrillation or pulseless ventricular tachycardia persists after two defibrillation attempts.^3^ ^5^ ^6^ ^7^ Resuscitation guidelines from the United Kingdom and Europe recommend epinephrine only after three defibrillation attempts have been unsuccessful.^8^ ^9^

Use of epinephrine for cardiac arrest remains controversial,^7^ ^10^ ^11^ ^12^ ^13^ ^14^ ^15^ ^16^ ^17^ and it is not recommended as a first line treatment for cardiac arrest due to a shockable rhythm because immediate defibrillation is highly effective in achieving return of spontaneous circulation for most patients with ventricular fibrillation or pulseless ventricular tachycardia.^4^ ^5^ ^9^ Despite this, one Get With The Guidelines-Resuscitation (GWTG-R) study found that 51% of patient with in-hospital cardiac arrest with an initial shockable rhythm that was refractory to first defibrillation within two minutes were treated with epinephrine before the second defibrillation, contrary to current guidelines. Treatment with epinephrine in these patients was associated with 30% lower odds of survival.^18^ The extent to which use of epinephrine occurs before a first defibrillation in patients with in-hospital cardiac arrest due to a shockable rhythm and the association of this approach with outcomes has not yet been examined.

To deal with this gap in knowledge, we used data from the GWTG-R registry, a large, multicenter registry of in-hospital cardiac arrest in the US, to examine the frequency of use of epinephrine before first defibrillation in patients with a shockable in-hospital cardiac arrest; and the association of epinephrine before defibrillation with survival to discharge, favorable neurological survival, and survival after acute resuscitation.

## Methods

### Study design and setting

We used 2000-18 data from the American Heart Association’s GWTG-R registry—a large, prospective, multisite registry of in-hospital cardiac arrest in the US. The design of this registry has been previously described.^19^ Hospitals participating in the registry submit clinical information about the medical history, hospital care, and outcomes of consecutive patients admitted to hospital for cardiac arrest using an online, interactive case report form and patient management tool (IQVIA, Parsippany, NJ). All patients with a confirmed in-hospital cardiac arrest, defined as absence of a palpable central pulse, apnea, and unresponsiveness and without do not resuscitate orders, are enrolled by trained personnel at participating hospitals. Many approaches to finding cases are used, including review of a centralized collection of cardiac arrest flow sheets, routine checks of code cards, pharmacy tracer drug records, reviews of hospital paging system logs, and hospital billing charges for resuscitation medication. Hospital participation is voluntary, with data collected using standardized “Utstein-style” definitions for all patient variables and outcomes to facilitate uniform reporting across hospitals.^20^ ^21^ To ensure accuracy and completeness of the data, rigorous training and certification of the medical staff at participating hospitals is carried out, standardized software is used with internal checks, and periodic re-abstractions and audits of collected data are made. The study is reported in accordance with the Strengthening the Reporting of Observational Studies in Epidemiology (STROBE) guidelines.^22^

### Study cohort

**Fig 1:**
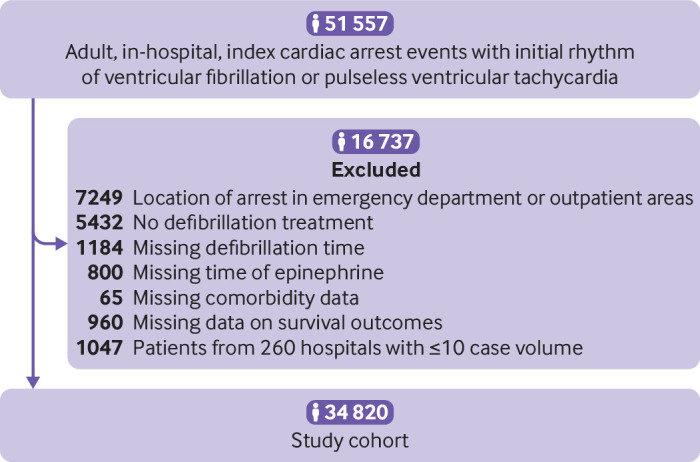
Study cohort. The flow chart shows the derivation of the study cohort after applying the study’s inclusion and exclusion criteria, and the number of participants excluded at each step

We identified 51 557 adult patients aged 18 or older with an index in-hospital cardiac arrest due to an initial rhythm of ventricular fibrillation or pulseless ventricular tachycardia. We excluded 7249 patients who had an in-hospital cardiac arrest in the emergency department and outpatient areas to focus our study on in-hospital arrests and avoid the inclusion of patients with out-of-hospital cardiac arrest who might have had a second arrest after arrival at the emergency department. Patients were also excluded if they did not receive any defibrillation (n=5432) or data were missing on timing of defibrillation (1184) or epinephrine (n=800), on comorbidities (n=65), or on survival outcomes (n=960). Finally, we excluded 1047 patients at 260 hospitals who had a case volume of less than or equal to 10 during the study period to ensure that our findings were not due to the inclusion of hospitals with very low volumes and limited experience in treating shockable in-hospital cardiac arrest. Our final cohort included 34 820 patients at 497 hospitals (fig 1).

### Study variables and outcomes

The primary exposure was administration of epinephrine before first defibrillation. For each patient, we calculated the time intervals (in minutes) from the onset of cardiac arrest to the administration of epinephrine and first defibrillation, respectively, which were used to determine whether patients were treated with epinephrine first or defibrillation first. Patients with identical administration times for epinephrine and defibrillation were included in the defibrillation first group in the primary analysis.

The primary outcome was survival to discharge. Given that both epinephrine and defibrillation are used for achieving return of spontaneous circulation, we also examined survival after acute resuscitation, defined as return of spontaneous circulation for at least 20 minutes. Finally, to determine whether difference in survival was clinically important, we also examined neurological disability using the cerebral performance category scores^23^: a score of 1 denoting mild or no neurological disability; 2, moderate neurological disability; 3, severe neurological disability, 4, coma or vegetative state; and 5, brain death. Favorable neurological survival was defined as survival with a cerebral performance category score of 1 or 2. Discharge scores were missing in 7.5% of patients, and thus analyses after discharge were limited to patients with available discharge cerebral performance category scores.

Patient data from the GWTG-R registry included demographic variables (age, sex, race), pre-existing conditions (current or previous myocardial infarction, current or previous heart failure, diabetes mellitus, renal insufficiency, hepatic insufficiency, respiratory insufficiency, pneumonia, sepsis, hypotension, acute stroke, baseline central nervous system depression, major trauma, metastatic or hematologic malignancy, metabolic or electrolyte abnormalities), cardiac arrest characteristics (initial rhythm (ventricular fibrillation or pulseless ventricular tachycardia)), location of arrest (intensive care unit, operating room, procedural area, telemetry unit, ward without telemetry), activation of a hospital-wide response, time of day (daytime: 7 am-11 pm, night-time: 11 pm-7 am), and calendar year, and interventions in place immediately before arrest (mechanical ventilation, antiarrhythmic drugs, dialysis, and intravenous vasoactive drugs). Hospital variables included total number of admissions, total inpatient beds, geographic census region (North and Mid-Atlantic, South Atlantic, North Central, South Central, Mountain, and Pacific), ownership (military, non-profit, government, private), and teaching status (major teaching, minor teaching, non-teaching). An “unknown” category was included for hospitals with missing data. Timely defibrillation was defined as defibrillation within two minutes of onset of in-hospital cardiac arrest and was evaluated as a potential explanatory variable in the association between early epinephrine and survival.

### Statistical analysis

Given the large sample size, we compared baseline patient and hospital characteristics between our exposure groups using standardized differences.^24^ Next, we compared the unadjusted rates of study outcomes in patients who did and did not receive epinephrine before first defibrillation, and compared the differences between the two groups using Student’s t tests. To account for treatment selection bias between patients who received epinephrine before defibrillation and patients who received defibrillation as the first treatment of cardiac arrest, we conducted a time dependent, propensity score matched analysis to evaluate the independent association of epinephrine before defibrillation with survival outcomes. This approach has been used in previous studies of time dependent cardiac interventions and is designed to account for the fact that epinephrine would not be given to patients in whom return of spontaneous circulation had already occurred or resuscitation had been terminated.^18^ ^25^ ^26^ Propensity scores were estimated using a non-parsimonious multivariable logistic regression model, where the dependent variable was whether a patient received epinephrine before defibrillation. All patient covariates listed in the section “Study variables” were included in the model. 

After estimation of propensity scores from the above model, we conducted a 1:1 risk set matching on the propensity score using nearest neighbor matching algorithm with a caliper width of 20% of the standard deviation of the logit of the propensity score. Patients who received epinephrine during a given minute of resuscitation after the onset of cardiac arrest (eg, 0, 1, 2) were propensity matched with a similar patient who was “at risk” of receiving epinephrine during the same minute (that is, no return of spontaneous circulation and still undergoing resuscitation) and had not yet received epinephrine. This approach ensured that patients in the groups receiving epinephrine were not inadvertently matched with patients who had already achieved return of spontaneous circulation and therefore “not at risk” of receiving epinephrine, which would have biased our study towards finding a negative association of epinephrine with survival outcomes. Thus, our propensity matched pairs comprised a patient who had received epinephrine at a given minute of resuscitation with a patient who had not yet received epinephrine within that same minute but had a similar probability of receiving it based on measured variables and had also not achieved return of spontaneous circulation (fig S2).

To evaluate the success of matching, we calculated standardized differences between matched patients, and considered a standardized difference of less than 10% as indicative of successful matching.^24^ Within our matched sample, we used conditional logistic regression to determine the association of epinephrine before defibrillation with study outcomes. In these analyses, we used clustered standard errors to account for correlation in the data if participants selected as controls were subsequently selected as patients if they received epinephrine at a later minute. Statistical significance was set at α=0.05. All statistical analysis was performed in SAS (version 9.4, SAS Institute, Cary, NC). The primary propensity matched analysis included only patient variables. In a secondary analysis, we also included hospital variables in the propensity score estimation algorithm.

### Sensitivity analysis

We conducted a range of sensitivity analyses to evaluate the robustness of our findings. Firstly, we repeated our primary analysis after excluding patients who received epinephrine and defibrillation during the same minute. Secondly, to determine whether the association of epinephrine with survival was due to a direct effect of epinephrine or due to a delay in defibrillation, we repeated the primary analysis after also matching patients according to defibrillation time in addition to the propensity scores. Thirdly, we conducted an additional analysis in which participants matched as controls with a patient who received epinephrine during a given minute were not eligible to be selected as subsequent patients if they received epinephrine during a later minute. Finally, we used inverse probability treatment weighting analyses to ensure that the association of epinephrine before defibrillation with study outcomes was consistent.

### Patient and public involvement

Since the study involved the use of deidentified data, patients were not involved in the design or conduct of the study.

## Results

**Fig 2:**
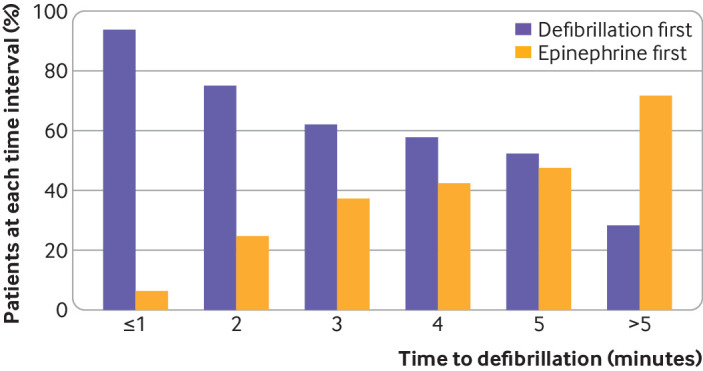
Proportion of participants receiving epinephrine (adrenaline) first or defibrillation first by time to defibrillation. The yellow bars represent the proportion of participants receiving epinephrine first and the purple bars represent the proportion of participants receiving defibrillation first (that is, without prior epinephrine). The figure shows that the proportion of patients receiving epinephrine first was associated with increasing delay in time to defibrillation

Of 34 820 patients, a total of 7054 (20.3%) received epinephrine before defibrillation. Table 1 compares patient characteristics stratified by whether epinephrine was administered before defibrillation or whether patients received defibrillation first. The group who received epinephrine before defibrillation were more likely to be black patients, have renal insufficiency, respiratory insufficiency, or sepsis, but less likely to have myocardial infarction during the admission to hospital than patients treated with defibrillation first. They were more likely to be receiving mechanical ventilation but less likely to be receiving intravenous antiarrhythmic drugs at the time of arrest (absolute standardized differences >10% for all). Most patients in both groups were in a monitored location (intensive care unit or telemetry unit), with only 11.4% located in a ward without telemetry. Overall, the median time to defibrillation was 1 minute (interquartile range 0-3), which was longer in the epinephrine first group than in the defibrillation first group (median 4 minutes (2-8) *v* 0 minutes (0-1); table 1 and fig 2). In our cohort, the proportion of patients treated with epinephrine before defibrillation increased over time, from 13.1% in 2000 to 22.3% in 2018 (P for trend <0.001; supplementary fig S1). The differences in magnitude of hospital characteristics between exposure groups were small (table 2). 

**Table 1:** Baseline characteristics of study patients overall and stratified by patients receiving epinephrine (adrenaline) first and defibrillation first

Variable	Overall (n=34 820)	Epinephrine first (n=7054)	Defibrillation first (n=27 766)	Standardized difference	
Personal characteristics:					
Age (years):					
Mean (SD)	65.8 (14.4)	65.6 (15.0)	65.9 (14.3)	2.3	
Median (IQR)	67 (57-77)	67 (57-77)	67 (57-77)		
Sex:				−1.9	
Male	22 039 (63.3)	4412 (62.5)	17 627 (63.5)		
Female	12 781 (36.7)	2642 (37.5)	10 139 (36.5)		
Race					
White	26 531 (76.2)	5093 (72.2)	21 438 (77.2)	−11.5	
Black	4972 (14.3)	1274 (18.1)	3698 (13.3)	13.1	
Other	3317 (9.5)	687 (9.7)	2630 (9.5)	0.1	
Hispanic ethnic origin	1597 (4.6)	412 (5.8)	1185 (4.3)	7.2	
Pre-existing conditions					
Myocardial infarction, this admission	9725 (27.9)	1390 (19.7)	8335 (30.0)	−24.0	
Myocardial infarction, previous admission	7947 (22.8)	1393 (19.7)	6554 (23.6)	−9.4	
Congestive heart failure, this admission	7642 (21.9)	1415 (20.1)	6227 (22.4)	−5.8	
Congestive heart failure, previous admission	9095 (26.1)	1806 (25.6)	7289 (26.3)	−1.5	
Diabetes mellitus	11 432 (32.8)	2470 (35.0)	8962 (32.3)	5.8	
Renal insufficiency	10 753 (30.9)	2610 (37.0)	8143 (29.3)	16.4	
Hepatic insufficiency	2055 (5.9)	485 (6.9)	1570 (5.7)	5.0	
Respiratory insufficiency	12 292 (35.3)	2894 (41.0)	9398 (33.8)	14.9	
Pneumonia	3482 (10.0)	838 (11.9)	2644 (9.5)	7.6	
Sepsis/septicemia	4021 (11.5)	1108 (15.7)	2913 (10.5)	15.5	
Hypotension/hypoperfusion	7991 (22.9)	1791 (25.4)	6200 (22.3)	7.2	
Acute stroke	1265 (3.6)	287 (4.1)	978 (3.5)	2.9	
Acute CNS event non-stroke	1865 (5.4)	407 (5.8)	1458 (5.3)	2.3	
Baseline CNS depression	2742 (7.9)	597 (8.5)	2145 (7.7)	2.7	
Major trauma	886 (2.5)	267 (3.8)	619 (2.2)	9.1	
Metastatic/hematologic malignancy	2814 (8.1)	727 (10.3)	2087 (7.5)	9.8	
Metabolic/electrolyte abnormalities	5428 (15.6)	1285 (18.2)	4143 (14.9)	8.9	
Cardiac arrest characteristics					
Initial cardiac rhythm				−6.3	
Pulseless ventricular fibrillation	21 539 (61.9)	4237 (60.1)	17 302 (62.3)		
Pulseless ventricular tachycardia	13 281 (38.1)	2817 (39.9)	10 464 (37.7)		
Time to first defibrillation (min)					
0 to <2	22 288 (64.0)	1376 (19.5)	20 912 (75.3)	−134.8	
>2 to 4	6042 (17.4)	1780 (25.2)	4262 (15.4)	−113.7	
>4	6490 (18.6)	3898 (55.3)	2592 (9.3)	112.7	
Location of arrest:					
Intensive care unit	19 094 (54.8)	3956 (56.1)	15 138 (54.5)	3.1	
Operating room/procedural area	4144 (11.9)	709 (10.1)	3435 (12.4)	−7.4	
Inpatient ward with telemetry	7121 (20.5)	1276 (18.1)	5845 (21.1)	−7.5	
Inpatient ward without telemetry	3969 (11.4)	1026 (14.5)	2943 (10.6)	11.9	
Other	492 (1.4)	87 (1.2)	405 (1.5)	−2.0	
Activation of hospital-wide response	27 283 (78.4)	5532 (78.4)	21 751 (78.3)	0.2	
Night-time arrest (11 pm to 7 am)	10 219 (29.4)	2233 (31.7)	7986 (28.8)	6.3	
Interventions in place at the time of arrest:					
Assisted or mechanical ventilation	12 727 (36.6)	2935 (41.6)	9792 (35.3)	13.1	
Antiarrhythmic agent	2771 (8.0)	325 (4.6)	2446 (8.8)	−16.9	
Dialysis or extracorporeal treatments	1195 (3.4)	300 (4.3)	895 (3.2)	5.4
Vasoactive agent	9397 (27.0)	2028 (28.7)	7369 (26.5)	4.5	
Calendar year of arrest:					
2000-05	9871 (28.3)	1481 (21.0)	8390 (30.2)	−21.2	
2006-10	9009 (25.9)	1791 (25.4)	7218 (26.0)	−1.4	
2011-15	9252 (26.6)	2158 (30.6)	7094 (25.6)	11.2	
2016-18	6688 (19.2)	1624 (23.0)	5064 (18.2)	11.8	

CNS=central nervous system; IQR=interquartile range; SD=standard deviation.

**Table 2:** Hospital characteristics overall and stratified by patients receiving epinephrine (adrenaline) first and defibrillation first

Variable	Overall (n=34 820)	Epinephrine first (n=7054)	Defibrillation first (n=27 766)	Standardized difference
Total No of admissions:				
100-2499	222 (0.6)	51 (0.7)	171 (0.6)	1.3
2500-4999	453 (1.3)	92 (1.3)	361 (1.3)	0.0
5000-7499	1178 (3.4)	213 (3.0)	965 (3.5)	−2.6
7500-9999	1345 (3.9)	274 (3.9)	1071 (3.9)	0.1
10 000-14 999	4747 (13.6)	930 (13.2)	3817 (13.8)	−1.6
15 000-19 999	6124 (17.6)	1298 (18.4)	4826 (17.4)	2.7
20 000-29 999	7122 (20.5)	1362 (19.3)	5760 (20.7)	−3.6
30 000-39 999	7345 (21.1)	1473 (20.9)	5872 (21.1)	−0.7
≥40 000	4360 (12.5)	916 (13.0)	3444 (12.4)	1.7
Unknown	1924 (5.5)	445 (6.3)	1479 (5.3)	4.2
Total No of inpatient beds:				
1-99	542 (1.6)	112 (1.6)	430 (1.5)	0.3
100-199	2318 (6.7)	438 (6.2)	1880 (6.8)	−2.3
200-249	1914 (5.5)	351 (5.0)	1563 (5.6)	−2.9
250-299	2608 (7.5)	533 (7.6)	2075 (7.5)	0.3
300-349	2822 (8.1)	558 (7.9)	2264 (8.2)	−0.9
350-499	7877 (22.6)	1579 (22.4)	6298 (22.7)	0.7
≥500	14 815 (42.6)	3038 (43.0)	11 777 (42.4)	1.3
Unknown	1924 (5.5)	445 (6.3)	1479 (5.3)	4.2
Geographic census region:				
North and Mid-Atlantic	4834 (13.9)	1032 (14.6)	3802 (13.7)	2.7
South Atlantic	8435 (24.2)	1671 (23.7)	6764 (24.4)	−1.6
North Central	8036 (23.1)	1483 (21.0)	6553 (23.6)	−6.2
South Central	6445 (18.5)	1435 (20.3)	5010 (18.0)	5.8
Mountain and Pacific	5540 (15.9)	1066 (15.1)	4474 (16.1)	−2.8
Unknown	1530 (4.4)	367 (5.2)	1163 (4.2)	4.8
Hospital ownership:				
Military	516 (1.5)	113 (1.6)	403 (1.5)	1.2
Non-profit	25 369 (72.9)	4972 (70.5)	20 397 (73.5)	−6.6
Government	2621 (7.5)	580 (8.2)	2041 (7.4)	3.3
Private	3040 (8.7)	636 (9.0)	2404 (8.7)	1.3
Unknown	3274 (9.4)	753 (10.7)	2521 (9.1)	5.3
Academic training program status				
Major teaching	11 523 (33.1)	2538 (36.0)	8985 (32.4)	7.6
Minor teaching	17 843 (51.2)	3449 (48.9)	14 394 (51.8)	−5.9
Non-teaching	3888 (11.2)	691 (9.8)	3197 (11.5)	−5.6
Unknown	1566 (4.5)	376 (5.3)	1190 (4.3)	4.9

Overall, 14 520 (41.7%) patients survived to hospital discharge, 11 595 (33.3%) had favorable neurological survival, and 26 602 (76.4%) survived after acute resuscitation. In unadjusted analysis, patients who received epinephrine before defibrillation were less likely to survive to hospital discharge (22.4% *v* 46.6%; difference −24.2%, 95% confidence interval −25.3% to −23.0%), have favorable neurological survival (15.9% *v* 37.9%; difference −22.0%, −23.1% to −21.0%), or survive after acute resuscitation (61.7% *v* 80.1%; difference −18.4%, −19.7% to −17.2%) than patients who were treated with defibrillation first.

To account for underlying differences in patients who did and did not receive epinephrine before defibrillation, we propensity matched a total of 13 138 patients (6569 in each group) for analyses of survival to discharge and survival after acute resuscitation. The distribution of propensity scores in the two groups is shown in figure S2 and table S1. Matching was successful in achieving covariate balance as shown by a standardized difference of <10% for all variables (fig S3; tables S2 and S3). In time dependent, propensity matched analysis, epinephrine before defibrillation was associated with decreased odds of survival to hospital discharge (odds ratio 0.69; 95% confidence interval 0.64 to 0.74), and survival after acute resuscitation (0.73; 0.67 to 0.79; table 3). For analyses of favorable neurological survival, a total of 12 486 patients (6243 in each group) were propensity matched. Epinephrine before defibrillation was associated with decreased odds of favorable neurological survival (odds ratio 0.68; 95% confidence interval 0.61 to 0.76). The negative association of epinephrine with study outcomes was consistent in secondary analysis, which also included hospital characteristics in the propensity score model (table 3).

**Table 3:** Association of epinephrine (adrenaline) before defibrillation with survival outcomes

Outcome	Epinephrine first	Defibrillation first	Adjusted OR (95% CI)
Without hospital characteristics (n=6569):			
Survival to hospital discharge	1471 (22.4)	1949 (29.7)	0.69 (0.64 to 0.74)
Survival after acute resuscitation	4055 (61.7)	4568 (69.5)	0.73 (0.67 to 0.79)
Favorable neurological survival*	985 (15.8)	1348 (21.6)	0.68 (0.61 to 0.76)
With hospital characteristics (n=6133):			
Survival to hospital discharge	1378 (22.5)	1730 (28.2)	0.74 (0.68 to 0.80)
Survival after acute resuscitation	3794 (61.9)	4243 (69.2)	0.72 (0.67 to 0.78)
Favorable neurological survival†	931 (15.9)	1190 (20.4)	0.74 (0.66 to 0.83)

OR=odds ratio.

* Sample size for analysis was 12 486 (6243 matched pairs).

† Sample size for analysis was 11 690 (5845 matched pairs).

### Sensitivity analyses

When the 5592 (16.1%) patients receiving epinephrine and defibrillation at the same minute were excluded, the associations between epinephrine and survival to discharge (odds ratio 0.74, 95% confidence interval 0.67 to 0.82) and survival after acute resuscitation (0.75, 0.68 to 0.83) were similar to the main analysis (table S4).

**Fig 3:**
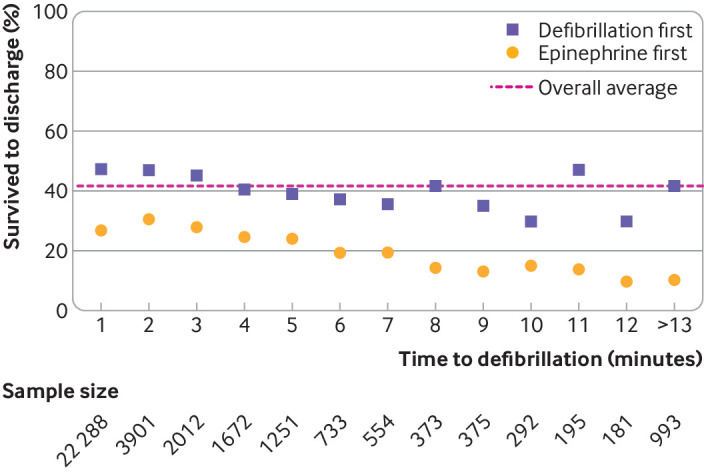
Rates of survival in patients receiving epinephrine (adrenaline) first versus defibrillation first stratified according to the time to defibrillation. The yellow circles represent survival in participants receiving epinephrine first and the purple squares represent survival in participants who received defibrillation first. The pink dashed line represents the overall survival rate in the entire cohort. The figure shows that in comparison with survival in participants receiving defibrillation first, survival in patients receiving epinephrine first was lower regardless of time to defibrillation

Given that patients who received epinephrine before defibrillation were also more likely to have delayed defibrillation, we conducted additional analyses to explore the extent to which the negative association of epinephrine with survival was due to delays in defibrillation. Stratified by each minute of defibrillation time, unadjusted survival was consistently lower in patients who received epinephrine before defibrillation than in patients who received defibrillation without prior epinephrine (fig 3). In propensity score analyses in which we additionally matched patients according to defibrillation time, the association of epinephrine with lower survival to hospital discharge (odds ratio 0.82; 95% confidence interval 0.73 to 0.93) and survival after acute resuscitation (0.78; 0.70 to 0.88) was consistent with the main analysis (table S5). Study findings were consistent when participants were not allowed to be selected as both controls and cases (table S6). Finally, similar findings were noted when we evaluated the association of epinephrine before defibrillation using inverse probability of treatment weighting (table S7).

## Discussion

### Principal findings

In this study of 34 820 patients with in-hospital cardiac arrest due to an initial shockable rhythm, we found that one in five patients receive epinephrine before defibrillation, contrary to resuscitation guidelines, which prioritize immediate defibrillation as the first line treatment.^4^ ^8^ ^9^ Epinephrine before defibrillation was associated with lower odds of survival to discharge, favorable neurological survival, and survival after acute resuscitation in comparison with patients who received defibrillation first. The above associations were consistent across a range of sensitivity analyses, including analyses in which patients were matched according to defibrillation time, which suggests that the association of epinephrine before defibrillation with study outcomes was not entirely explained by delayed defibrillation in patients who were treated with epinephrine first. Several of our findings merit further consideration.

### Implications of study for in-hospital resuscitation care

For patients with in-hospital cardiac arrest due to a shockable rhythm, immediate defibrillation is highly effective, and delays in defibrillation are associated with lower survival rates.^27^ ^28^ The American Heart Association recommends that patient with in-hospital cardiac arrest with an initial shockable rhythm receive defibrillation within two minutes of cardiac arrest, whereas epinephrine is recommended only if the patient remains refractory to at least two defibrillation attempts (three defibrillation attempts according to UK and European guidelines).^8^ ^9^ Accordingly, the GWTG-R registry has adopted time to defibrillation within two minutes in patients with a shockable in-hospital cardiac arrest as a key hospital resuscitation quality metric and has devoted considerable effort and resources towards reducing delays in defibrillation in patients with a shockable rhythm.

Despite these efforts, it is noteworthy that roughly 20% of patients with in-hospital cardiac arrest with a shockable rhythm in GWTG-R were treated with epinephrine first instead of defibrillation. Our study found this practice was associated with worse survival to discharge and less favorable neurological survival, largely owing to lower odds of survival after acute resuscitation. Importantly, use of epinephrine was also associated with substantial delays in defibrillation, which is the primary treatment for such patients. These findings are concerning because, even though shockable rhythms comprise <20% of all in-hospital cardiac arrests, the likelihood of survival in patients with a shockable rhythm is three- to fourfold higher than for patients with a non-shockable rhythm, especially when defibrillation treatment can be provided without delay.^29^ ^30^ Efforts that prioritize defibrillation treatment and discourage epinephrine during the initial resuscitation of a patient with a shockable in-hospital cardiac arrest are urgently needed.

### Strengths and weaknesses in relation to other studies

To our knowledge, this is the first study to examine use of epinephrine before first defibrillation in patients with in-hospital cardiac arrest and adds to the growing evidence of the potential harm of early epinephrine for shockable in-hospital cardiac arrest. Our findings are consistent with a previous GWTG-R study that found worse survival in patient with in-hospital cardiac arrest with ventricular fibrillation or pulseless ventricular tachycardia refractory to first defibrillation who were treated with epinephrine before second defibrillation (odds ratio 0.70; 95% confidence interval 0.59 to 0.82; P<0.001).^18^ Only patients who remained in ventricular fibrillation after first shock were included in that study, however. Such patients are a small proportion of patients with a shockable in-hospital cardiac arrest and require entirely different care. Our study highlights that the use of epinephrine before defibrillation is even more pervasive than previously shown.

Epinephrine might be detrimental to patients with a shockable cardiac arrest, which commonly has a primary cardiac cause (eg, acute myocardial infarction). Use of epinephrine could lead to increased demand for myocardial oxygen owing to its positive inotropic and chronotropic effects, and reduced blood flow to other organs, which might be associated with worse outcomes in these patients.^31^ ^32^ ^33^ ^34^ Prompt defibrillation is an effective treatment for ventricular fibrillation or pulseless ventricular tachycardia, but use of epinephrine first might contribute to delays in defibrillation. Indeed, we found that the median time to defibrillation was three minutes in the group receiving epinephrine first compared with zero minutes in the defibrillation group. Nevertheless, we found that the negative association of epinephrine with survival persisted even after matching according to defibrillation time in sensitivity analyses.

We found that several patient variables were associated with the use of epinephrine before defibrillation. In particular, we found that black and Hispanic patients were more likely to receive epinephrine before defibrillation than white patients, which is consistent with previous reports that showed an increased risk of poor quality resuscitation care among racial and ethnic minority patients.^35^ ^36^ Most patients who received epinephrine before defibrillation were located in a monitored setting (eg, intensive care unit, a ward with telemetry, or a procedural area), which suggests that the practice is widespread across hospitals. We also found that use of epinephrine before defibrillation was more common in sicker patients with more comorbidities. Our analytic approach considered these baseline differences in patient characteristics, and we achieved covariate balance using propensity scores. A notable strength of our study was that we used a time dependent, propensity score method to match patients who received epinephrine with similar patients who were still undergoing resuscitation and in whom return of spontaneous circulation had not yet been achieved. Thus our approach ensured that our study was not inherently biased toward finding a negative association of epinephrine with survival.

### Possible explanations and future directions

Several factors could explain why patients with ventricular fibrillation or pulseless ventricular tachycardia might be receiving epinephrine before defibrillation. Firstly, asystole and pulseless electrical activity comprise >85% of all in-hospital cardiac arrests in the US, for which early epinephrine is the recommended treatment. Recently, epinephrine within five minutes was adopted by GWTG-R as a quality metric for in-hospital cardiac arrest due to a non-shockable rhythm.^10^ ^37^ Possibly, the preponderance of non-shockable rhythms among patients with in-hospital cardiac arrest and the increased emphasis on timely epinephrine in such patients might have had a spillover effect from management of in-hospital cardiac arrest with a shockable rhythm. Secondly, nurses are usually the first responders when patients have a cardiac arrest. Although they are usually skilled in initiating cardiopulmonary resuscitation and administering drugs, they might be less inclined to perform defibrillation independently. Epinephrine might be given while waiting for the code team to arrive and perform defibrillation. Understanding the factors that explain the use of epinephrine before defibrillation in patients in hospital who arrest is an important next step, and will be crucial for designing hospital interventions to ensure that patients with shockable in-hospital cardiac arrest are treated with timely defibrillation instead of epinephrine first.

### Limitations 

Our findings should also be interpreted considering the following limitations. Firstly, the assignment of epinephrine first or defibrillation first was not random and there is potential for residual confounding from unmeasured patient variables. Secondly, data on time to epinephrine and time to defibrillation relied on reported times of cardiac arrest, epinephrine, and defibrillation, respectively, within hospital records. Possibly, a lack of synchronization between cardiac monitors and defibrillators, and the generally chaotic resuscitation environment, contributed to errors in measurement, leading to potential misclassification of exposure. Such misclassification is expected to be non-differential, however, and would bias our findings toward the null hypothesis as there is no reason to believe that misclassification of time in the same patient would be differential. Thirdly, our findings cannot establish causation, and it is possible that the use of epinephrine before defibrillation is a marker of lower quality resuscitation care delivery (eg, lower quality chest compressions or quality and experience of the first responders/code team), which is unmeasured. No randomized controlled trials on this treatment strategy are planned or ongoing, however, and such a trial would not be ethically feasible given the strong recommendation for immediate defibrillation in patients with a shockable rhythm. Therefore, despite our study’s limitations, we believe these are the best data on this topic. Finally, hospitals participating in the Get With The Guidelines-Resuscitation represent <5% of all acute care hospitals in the US, and therefore our findings might not be generalizable to non-participating hospitals. The Get With The Guidelines-Resuscitation represents hospitals that are committed to quality improvement of resuscitation, and use of epinephrine before defibrillation for shockable in-hospital cardiac arrest might be even more pervasive in non-participating hospitals.

### Conclusion

Contrary to resuscitation guidelines and protocols for adult cardiac life support, treatment with epinephrine before defibrillation is common and is associated with lower survival for in-hospital cardiac arrests due to a shockable rhythm.

**What is already known on this topic**

Guidelines recommend prompt defibrillation for treatment of in-hospital cardiac arrest due to an initial rhythm of ventricular fibrillation (or pulseless ventricular tachycardia)
Epinephrine (adrenaline) is recommended only when patients remain in refractory ventricular fibrillation or pulseless ventricular tachycardia after many defibrillation attempts

**What this study adds**

Despite strong guideline recommendations, one in five patients with in-hospital cardiac arrest due to ventricular fibrillation or pulseless ventricular tachycardia are treated with epinephrine before the first defibrillation
Use of epinephrine before defibrillation is associated with lower odds of survival to discharge and of favorable neurological survival, probably due to lower odds of achieving return of spontaneous circulation
The negative association of epinephrine with survival was not entirely explained by delayed defibrillation in patients who received epinephrine before first defibrillation

## Data Availability

Owing to the sensitive nature of the data collected for this study, requests to access the dataset may be obtained by qualified researchers trained in human subject confidentiality protocols after submitting a written request to the Get With The Guidelines-Resuscitation (GWTGResuscitationResearch@heart.org).

## Web extra

Extra material supplied by authors

Web appendix: Supplementary appendix

## Notes

Contributors: EE, PSC, and SG conceived the study. MBS, NM, NB, and MV-S contributed to the study design. MBS prepared and cleaned the data and conducted the statistical analysis. MV-S supervised the statistical analysis. EE, MBS, and SG took the lead in drafting the manuscript. All authors contributed to interpretation of the data, provided critical revisions to the manuscript, and approved the final draft. SG is the guarantor. SG had full access to all the data in the study and takes responsibility for the integrity of the data and the accuracy of the data analysis. The corresponding author attests that all listed authors meet authorship criteria and that no others meeting the criteria have been omitted.

SG affirms that this manuscript is an honest, accurate and transparent account of the study being reported; and that no important aspects of the study have been omitted; and that any discrepancies from the study as planned (and if relevant, registered) have been explained.

Dissemination to participants and related patient and public communities: As the personal identifying information of participants has been removed from the study dataset, it is not possible to send the results of this study to participants. The study findings have been shared with the American Heart Association, which plans to disseminate these results upon publication to the participating hospitals through their newsletter.

Provenance and peer review: Not commissioned; externally peer reviewed.
