# Epinephrine before defibrillation in patients with shockable in-hospital cardiac arrest: propensity matched analysis

**DOI:** 10.1136/bmj.o2705

**Published:** 2022-11-18

In this paper by Evans and colleagues (*BMJ* 2021;375:e066534, doi:10.1136/bmj-2021-066534, published 10 November 2021), the authors reported that 27.7% of patients with an in-hospital cardiac arrest due to a shockable rhythm were treated with epinephrine before defibrillation, a practice that is contrary to the recommendations from the Advanced Cardiac Life Support guidelines. Use of epinephrine before defibrillation was associated with lower risk of survival outcomes.

During a follow-up study, the authors discovered that the estimate of 27.7% was incorrect. The correct proportion of patients who received epinephrine before defibrillation was 20.3%. The authors defined use of epinephrine before defibrillation if the time interval between onset of arrest and time of epinephrine administration was less than the time interval of onset of arrest and time of defibrillation. However, they learned that 2576 patients with identical epinephrine and defibrillation time were inadvertently misclassified as epinephrine first (exposed group). This error occurred because of the unique way SAS stores very large numerical values. Date/time variables often represent large numerical values as they are stored as the number of seconds since 1 January 1960, 0:00:00 hours. To handle large values, SAS uses 64-bit floating point arithmetic. A detailed exposition of the floating point arithmetic feature in SAS is published elsewhere.^1^ ^2^ As outlined in these references, it is possible that two identical times (as represented by numerical conversions in SAS) differ by a very small fraction (eg, 0.000000477). As a result, mathematical operations to calculate time interval between onset of cardiac arrest and use of epinephrine and defibrillation, respectively, resulted in a non-zero (albeit very small) difference and misclassified 2576 patients to the group receiving epinephrine before defibrillation.

The authors have reanalysed their data and updated the manuscript. The reanalysis did not change the paper’s overall interpretation that epinephrine before defibrillation was associated with lower risk of survival outcomes. Corresponding author Saket Girotra was at the University of Iowa Carver College of Medicine when the original study was conducted but is now affiliated with the University of Texas Southwestern Medical Center, Dallas, TX, USA.
