# Supplementary material for: Epinephrine before defibrillation in patients with shockable in-hospital cardiac arrest: propensity matched analysis
Source: BMJ. 2021 Nov 10;375:e066534. doi: 10.1136/bmj-2021-066534 (PMC8579224; doi:10.1136/bmj-2021-066534)
Supplement: Supplementary file 1 — Web appendix: Supplementary appendix [file evae066534.ww.pdf]

Supplementary Appendix

Epinephrine Before Defibrillation in Patients with Shockable In-hospital Cardiac Arrest: A Propensity Matched Analysis

for the American Heart Association's Get With the Guidelines®-Resuscitation Investigators

Erin Evans MD *critical care fellow*; Morgan B. Swanson BS *medical student and PhD candidate*; Nicholas Mohr MD, MS *professor of emergency medicine, epidemiology, and anaesthesia-critical care*; Nassar Boulos MD *clinical associate professor of medicine*; Mary Vaughan-Sarrazin PhD *research associate professor of medicine*; Paul S. Chan MD, MSc *professor of medicine*; Saket Girotra MD, SM *associate professor of medicine*

Besides the study authors Paul S. Chan MD, MSc and Saket Girotra MD, SM, the Get With the Guidelines®-Resuscitation Investigators include Anne Grossestreuer PhD; Ari Moskowitz MD; Dana Edelson MD MS; Joseph Ornato MD; Mary Ann Peberdy MD; Matthew Churpek MD MPH PhD; Michael Kurz MD MS-HES; Monique Anderson Starks MD MHS; Sarah Perman MD MSCE; Zachary Goldberger MD MS

Supplementary Figure Legends

Figure S1. Calendar-Year Trends in the Proportion of Patients With an Initial Shockable Rhythm Treated With Epinephrine Before Defibrillation. The proportion of patients with an initial rhythm of ventricular fibrillation or pulseless ventricular tachycardia increased from 13.1% in 2000 to 22.3% in 2018 (P for trend < 0.001)

Figure S2. Distribution of Propensity Scores Across Study Exposure Groups. Panel A shows the distribution of propensity score in patients who received defibrillation first. Panel B shows the distribution of propensity scores in patients who received epinephrine first. In both panels, patients who were matched are represented by yellow bars, and patients who were not matched are represented by gray bars

Figure S3. Standardized Differences in Study Variables Before and After Matching. The grey circles represent the standardized difference for study variables before propensity matching and the black circles represent standardized difference after propensity matching. The dotted vertical lines represent the standardized difference of 10%. The figure shows that propensity score matching was successful in achieving covariate balance for all study variables (i.e., all standardized differences were <10%).

Figure S1

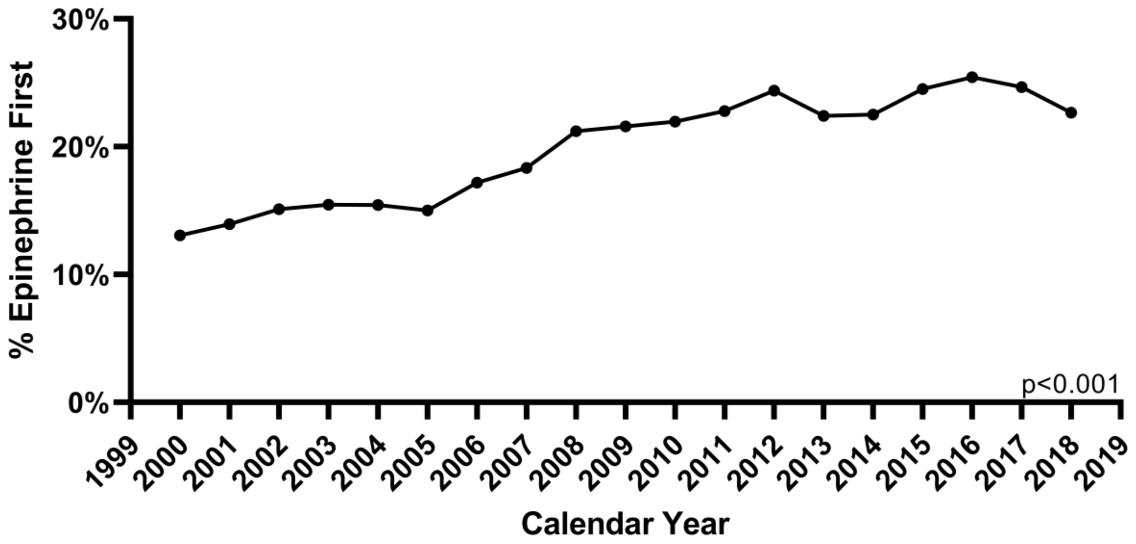
30%
20%
10%
0%
% Epinephrine First
p<0.001
1999
2000
2001
2002
2003
2004
2005
2006
2007
2008
2009
2010
2011
2012
2013
2014
2015
2016
2017
2018
2019
Calendar Year

Figure S2

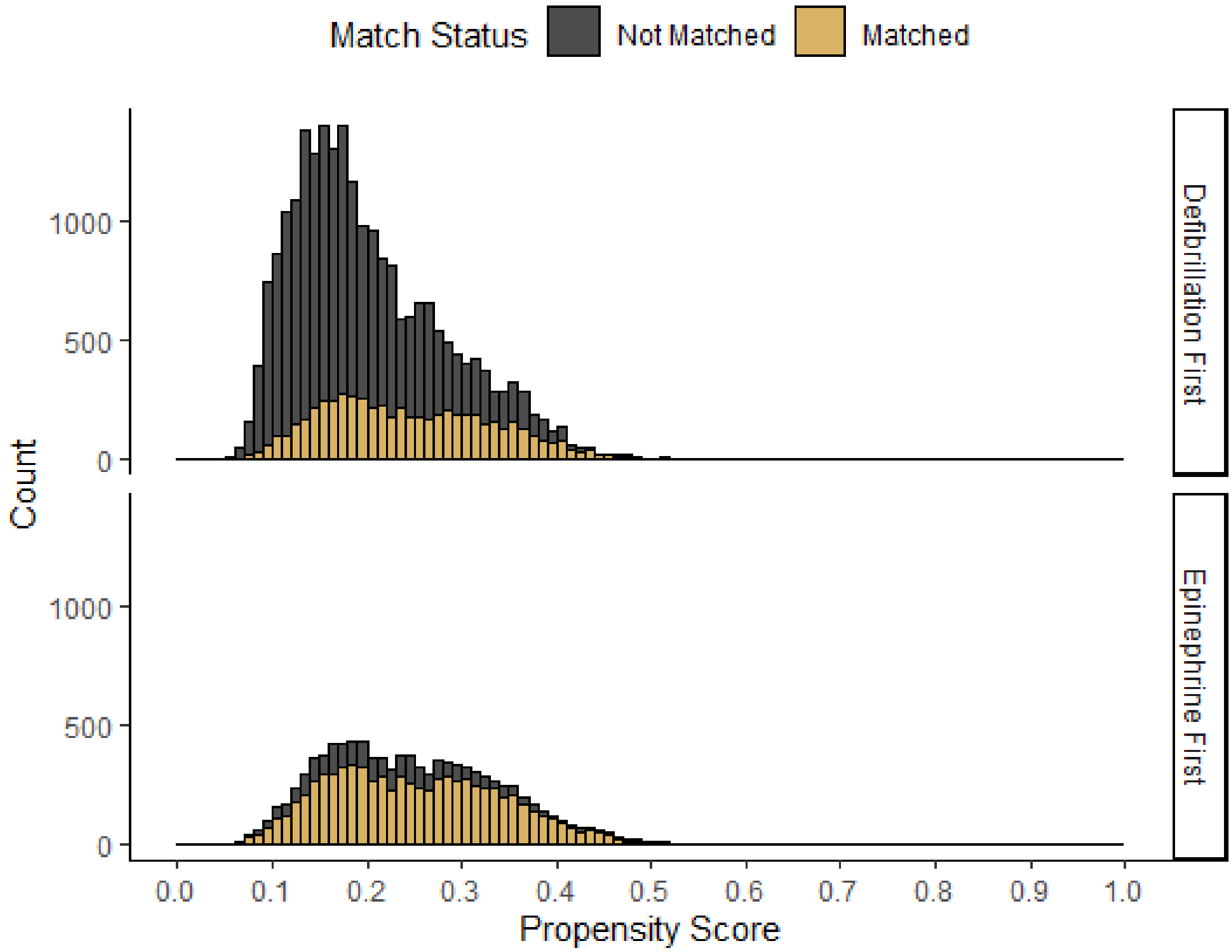

Match Status
Not Matched
Matched
Count
0
500
1000
0
500
1000
Defibrillation First
Epinephrine First
0.0
0.1
0.2
0.3
0.4
0.5
0.6
0.7
0.8
0.9
1.0
Propensity Score

Figure S3

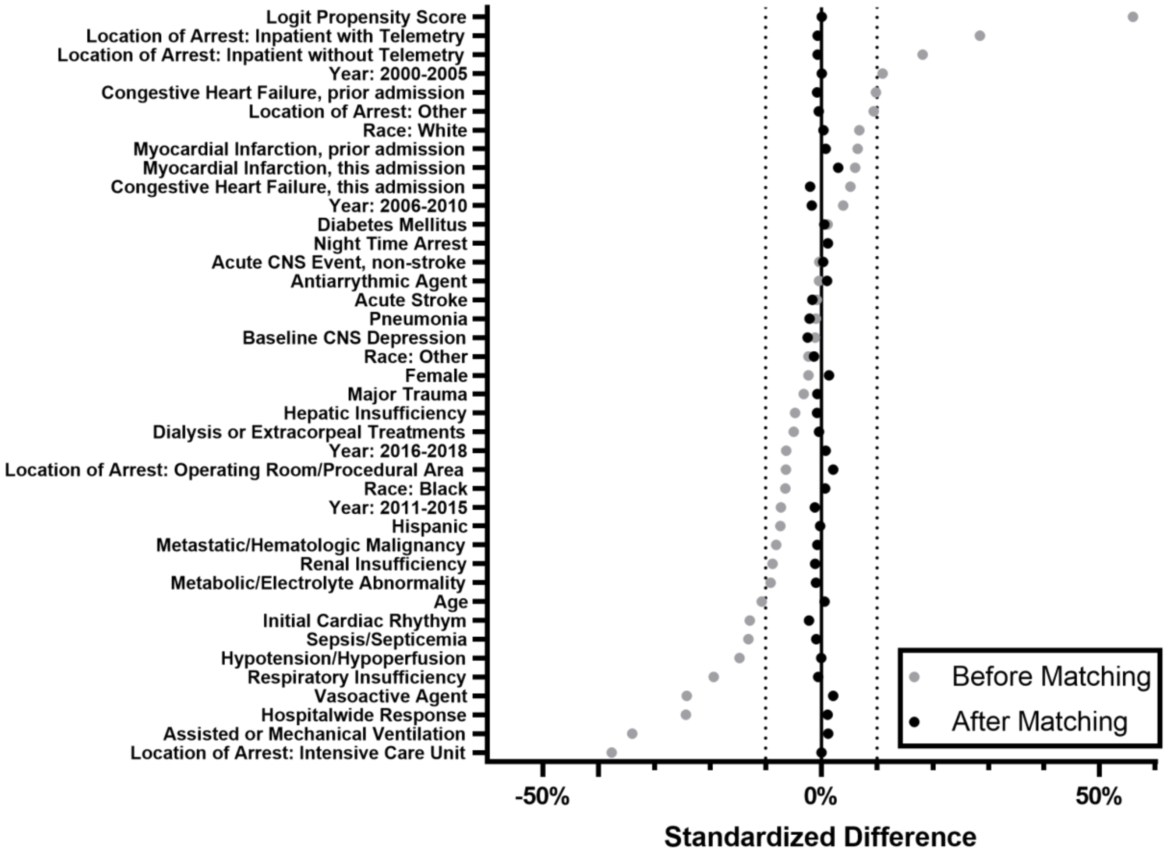

Logit Propensity Score
Location of Arrest: Inpatient with Telemetry
Location of Arrest: Inpatient without Telemetry
Year: 2000-2005
Congestive Heart Failure, prior admission
Location of Arrest: Other
Race: White
Myocardial Infarction, prior admission
Myocardial Infarction, this admission
Congestive Heart Failure, this admission
Year: 2006-2010
Diabetes Mellitus
Night Time Arrest
Acute CNS Event, non-stroke
Antiarrythmic Agent
Acute Stroke
Pneumonia
Baseline CNS Depression
Race: Other
Female
Major Trauma
Hepatic Insufficiency
Dialysis or Extracorpeal Treatments
Year: 2016-2018
Location of Arrest: Operating Room/Procedural Area
Race: Black
Year: 2011-2015
Hispanic
Metastatic/Hematologic Malignancy
Renal Insufficiency
Metabolic/Electrolyte Abnormality
Age
Initial Cardiac Rhythym
Sepsis/Septicemia
Hypotension/Hypoperfusion
Respiratory Insufficiency
Vasoactive Agent
Hospitalwide Response
Assisted or Mechanical Ventilation
Location of Arrest: Intensive Care Unit
-50%
0%
50%
Standardized Difference
Before Matching
After Matching

Table S1. Distribution of Propensity Scores

	Epinephrine First	Defibrillation First
**Mean (SD)**	0.25 (0.09)	0.24 (0.09)
**Median (IQR)**	0.24 (0.18 to 0.31)	0.24 (0.18 to 0.31)
**Range**	0.06 to 0.55	0.06 to 0.54

Table S2. Subject Demographics by Exposure Group Before and After Matching.

	Before Match		After Match		
Variable	Epinephrine First N=7,054	Defibrillation First N=27,766	Epinephrine First N=6,569	Defibrillation First N=6,569	Standardized Difference
Demographics					
Age (years), mean (SD)	65.6 (15.0)	65.9 (14.3)	65.7 (15.0)	65.6 (14.8)	-0.8
Median (IQR)	67 (57 – 77)	67 (57 – 77)	67 (57 -77)	67 (57 -77)	
Sex (n, %)					0.8
Female	2,642 (37.5%)	10,139 (36.5%)	2,461 (37.5%)	2,488 (37.9%)	
Male	4,412 (62.5%)	17,627 (63.5%)	4,108 (62.5%)	4,081 (62.1%)	
Race (n, %)					
White	5,093 (72.2%)	21,438 (77.2%)	4,764 (72.5%)	4,776 (72.7%)	0.4
Black	1,274 (18.1%)	3,698 (13.3%)	1,169 (17.8%)	1,144 (17.4%)	-1.0
Other	687 (9.7%)	2,630 (9.5%)	636 (9.7%)	649 (9.9%)	0.6
Hispanic Ethnicity (n,%)	412 (5.8%)	1,185 (4.3%)	384 (5.9%)	396 (6.0%)	0.8
Calendar Year of Arrest					
2000-2005	1,481 (21.0%)	8,390 (30.2%)	1,369 (20.8%)	1,351 (20.6%)	-0.6
2006-2010	1,791 (25.4%)	7,218 (26.0%)	1,699 (25.9%)	1,721 (26.2%)	0.8
2011-2015	2,158 (30.6%)	7,094 (25.6%)	1,999 (30.4%)	1,947 (29.6%)	-0.2
2016-2018	1,624 (23.0%)	5,064 (18.2%)	1,502 (22.9%)	1,550 (23.6%)	0.2
Pre-existing conditions (n, %)					
Current Myocardial Infarction	1,390 (19.7%)	8,335 (30.0%)	1,286 (19.6%)	1,279 (19.5%)	-0.3
Prior Myocardial Infarction	1,393 (19.7%)	6,554 (23.6%)	1,301 (19.8%)	1,244 (18.9%)	-2.2
Current Heart Failure	1,415 (20.1%)	6,227 (22.4%)	1,322 (20.1%)	1,304 (19.9%)	-0.7
Prior Heart Failure	1,806 (25.6%)	7,289 (26.3%)	1,693 (25.8%)	1,671 (25.4%)	-0.8
Diabetes Mellitus	2,470 (35.0%)	8,962 (32.3%)	2,302 (35.0%)	2,330 (35.5%)	0.9
Renal Insufficiency	2,610 (37.0%)	8,143 (29.3%)	2,443 (37.2%)	2,357 (35.9%)	-2.7
Hepatic Insufficiency	485 (6.9%)	1,570 (5.7%)	463 (7.1%)	476 (7.3%)	0.8
Respiratory Insufficiency	2,894 (41.0%)	9,398 (33.8%)	2,694 (41.0%)	2,635 (40.1%)	-1.8
Pneumonia	838 (11.9%)	2,644 (9.5%)	793 (12.1%)	758 (11.5%)	-1.7
Sepsis/Septicemia	1,108 (15.7%)	2,913 (10.5%)	1,040 (15.8%)	1,021 (15.5%)	-0.8
Hypotension/Hypoperfusion	1,791 (25.4%)	6,200 (22.3%)	1,657 (25.2%)	1,623 (24.7%)	-1.2
Acute Stroke	287 (4.1%)	978 (3.5%)	275 (4.2%)	272 (4.1%)	-0.2
Acute CNS Event, non-stroke	407 (5.8%)	1,458 (5.3%)	384 (5.9%)	398 (6.1%)	0.9
Baseline CNS Depression	597 (8.5%)	2,145 (7.7%)	551 (8.4%)	510 (7.8%)	-2.3
Major Trauma	267 (3.8%)	619 (2.2%)	252 (3.8%)	233 (3.6%)	-1.5
Malignancy	727 (10.3%)	2,087 (7.5%)	688 (10.5%)	643 (9.8%)	-2.3
Metabolic Abnormality	1,285 (18.2%)	4,143 (14.9%)	1,211 (18.4%)	1,193 (18.2%)	-0.7
Arrest Event Characteristics					
Initial Cardiac Rhythm (n, %)					2.2
Ventricular Fibrillation	4,237 (60.1%)	17,302 (62.3%)	3,693 (60.3%)	4,003 (60.9%)	
Ventricular Tachycardia	2,817 (39.9%)	10,464 (37.7%)	2,606 (39.7%)	2,566 (39.1%)	

Location of Arrest (n, %)					
Intensive Care Unit	3,956 (56.1%)	15,138 (54.5%)	3,685 (56.1%)	3,695 (56.3%)	0.3
Procedural Area	709 (10.1%)	3,435 (12.4%)	627 (9.5%)	654 (10.0%)	1.4
Telemetry Unit	1,276 (18.1%)	5,845 (21.1%)	1,198 (18.2%)	1,184 (18.0%)	-0.6
Non-telemetry Unit	1,026 (14.5%)	2,943 (10.6%)	980 (14.9%)	956 (14.6%)	-1.0
Other	87 (1.2%)	405 (1.5%)	79 (1.2%)	80 (1.2%)	0.1
Hospital-Wide Response (n, %)	5,532 (78.4%)	21,751 (78.3%)	5,181 (78.9%)	5,176 (78.8%)	-0.2
Night-time arrest (n, %)	2,233 (31.7%)	7,986 (28.8%)	2,088 (31.8%)	2,020 (30.8%)	-2.2
Interventions at Time of Arrest					
Mechanical Ventilation	2,935 (41.6%)	9,792 (35.3%)	2,705 (41.2%)	2,721 (41.4%)	0.5
Antiarrhythmic Agent	325 (4.6%)	2,446 (8.8%)	297 (4.5%)	298 (4.5%)	0.0
Dialysis	300 (4.3%)	895 (3.2%)	280 (4.3%)	280 (4.3%)	0.0
Vasoactive Agent	2,028 (28.7%)	7,369 (26.5%)	1,843 (28.1%)	1,860 (28.3%)	0.6

Table S3. Hospital Characteristics by Exposure Group Before and After Matching.

	Before Match		After Match		
**Variable**	**Epinephrine First N=7,054**	**Defibrillation First N=27,766**	**Epinephrine First N=6,569**	**Defibrillation First N=6,569**	**Standardized Difference**
Total Admissions					
100 – 2,499	51 (0.7%)	171 (0.6%)	49 (0.8%)	76 (1.2%)	4.2
2,500 – 4,999	92 (1.3%)	361 (1.3%)	83 (1.3%)	121 (1.8%)	4.7
5,000 – 7,499	213 (3.0%)	965 (3.5%)	194 (3.0%)	267 (4.1%)	6.0
7,500 – 9,999	274 (3.9%)	1,071 (3.9%)	250 (3.8%)	214 (3.3%)	-3.0
10,000 – 14,999	930 (13.2%)	3,817 (13.8%)	874 (13.3%)	896 (13.6%)	1.0
15,000 – 19,999	1,298 (18.4%)	4,826 (17.4%)	1,196 (18.2%)	1,093 (16.6%)	-4.1
20,000 – 29,999	1,362 (19.3%)	5,760 (20.7%)	1,274 (19.4%)	1,335 (20.3%)	2.3
30,000 – 39,999	1,473 (20.9%)	5,872 (21.1%)	1,381 (21.0%)	1,330 (20.3%)	-1.9
40,000+	916 (13.0%)	3,444 (12.4%)	841 (12.8%)	877 (13.4%)	1.6
Unknown	445 (6.3%)	1,479 (5.3%)	427 (6.5%)	360 (5.5%)	-4.3
Total Inpatient Beds					
1 – 99	112 (1.6%)	430 (1.5%)	104 (1.6%)	142 (2.2%)	4.3
100 – 199	438 (6.2%)	1,880 (6.8%)	399 (6.1%)	472 (7.2%)	4.5
200 – 249	351 (5.0%)	1,563 (5.6%)	332 (5.1%)	359 (5.5%)	1.8
250 – 299	533 (7.6%)	2,075 (7.5%)	502 (7.6%)	460 (7.0%)	-2.5
300 – 349	558 (7.9%)	2,264 (8.2%)	530 (8.1%)	538 (8.2%)	0.4
350 – 499	1,579 (22.4%)	6,298 (22.7%)	1,445 (22.0%)	1,399 (21.3%)	-1.7
500+	3,038 (43.0%)	11,777 (42.4%)	2,830 (43.1%)	2,839 (43.2%)	0.3
Unknown	445 (6.3%)	1,479 (5.3%)	427 (5.5%)	427 (6.5%)	-4.3
Geographic Region (n,					
North/Mid-Atlantic	1,032 (14.6%)	3,802 (13.7%)	965 (14.7%)	885 (13.5%)	-4.9
South Atlantic (incl.	1,671 (23.7%)	6,764 (24.4%)	1,564 (23.8%)	1,569 (23.9%)	-5.4
North Central	1,483 (21.0%)	6,553 (23.6%)	1,349 (20.5%)	1,505 (22.9%)	5.8
South Central	1,435 (20.3%)	5,010 (18.0%)	1,342 (20.4%)	1,284 (20.0%)	-2.2
Mountain/Pacific	1,066 (15.1%)	4,474 (16.1%)	996 (15.2%)	1,025 (15.6%)	1.2
Unknown	367 (5.2%)	1,163 (4.2%)	353 (5.4%)	301 (4.6%)	-3.6
Hospital Ownership (n,					
Military	113 (1.6%)	403 (1.5%)	103 (1.6%)	106 (1.6%)	0.4
Non-profit	4,972 (70.5%)	20,397 (73.5%)	4,626 (70.4%)	4,678 (71.2%)	1.7
Government	580 (8.2%)	2,041 (7.4%)	542 (8.3%)	524 (8.0%)	-1.0
Private	636 (9.0%)	2,404 (8.7%)	590 (9.0%)	564 (8.6%)	-1.4
Unknown	753 (10.7%)	2,521 (9.1%)	708 (10.8%)	697 (10.6%)	-0.5
Academic Training					
Major Teaching	2,538 (36.0%)	8,985 (32.4%)	2,363 (36.0%)	2,352 (35.8%)	-0.3
Minor Teaching	3,449 (48.9%)	14,394 (51.8%)	3,197 (48.7%)	3,203 (48.8%)	0.2
No Teaching	691 (9.8%)	3,197 (11.5%)	647 (9.9%)	710 (10.8%)	3.2
Unknown	376 (5.3%)	1,190 (4.3%)	362 (5.5%)	304 (4.6%)	-4.0

Table S4. Association of Epinephrine Before Defibrillation With Study Outcomes After Excluding Patients Receiving Both Treatments During the Same Minute

Outcome	Epinephrine First	Defibrillation First	Adjusted OR (95% CI)
	Without Hospital Characteristics		
	N=6,505	N=6,505	
Survival to Discharge	1,463 (22.5%)	1,827 (28.1%)	0.74 (0.67 to 0.82)
Acute Resuscitation Survival	4,014 (61.7%)	4,437 (68.2%)	0.75 (0.68 to 0.83)
Favorable Neurological Survival[a]	982 (15.9%)	1,248 (20.2%)	0.75 (0.66 to 0.84)

[a]Analyses for favorable neurological survival included a total of 12,338 patients (6,169 matched pairs)

Table S5. Association of Epinephrine Prior to Defibrillation After Matching on Time to Defibrillation

Outcome	Epinephrine First	Defibrillation First	Adjusted OR (95% CI)
	Without Hospital Characteristics		
	N=6,122	N=6,122	
Survival to Discharge	1,429 (23.3%)	1,653 (27.0%)	0.82 (0.73 to 0.93)
Acute Resuscitation Survival	3,790 (61.9%)	4,130 (67.5%)	0.78 (0.70 to 0.88)
Favorable Neurological Survival[a]	963 (16.6%)	1,135 (19.6%)	0.81 (0.71 to 0.94)

[a]Analyses for favorable neurological survival included a total of 11,598 patients (5,799 matched pairs)

Table S6. Association of Epinephrine Before Defibrillation With Study Outcomes using Propensity-matched Analysis Without Replacement of Controls

Outcome	Epinephrine First	Defibrillation First	Propensity-matched analysis without duplication
	Without Hospital Characteristics		
	N=5,506	N=5,506	
Survival to Discharge	1,291 (23.5%)	1,605 (29.2%)	0.72 (0.66 to 0.79)
Acute Resuscitation Survival	3,408 (61.9%)	3,782 (68.7%)	0.72 (0.66 to 0.78)
Favorable Neurological Survival[a]	870 (16.7%)	1,094 (21.1%)	0.72 (0.65 to 0.80)

[a]Analyses for favorable neurological survival included a total of 10,402 patients (5,201 matched pairs)

Table S7. Association of Epinephrine Before Defibrillation with Study Outcomes using Inverse Probability of Treatment Weighting Analysis.

	Inverse Propensity Score Weighted Outcome Measures (%)		
**Outcome**	**Epinephrine First N=5,718**	**Defibrillation First N=22,785**	**Adjusted OR (95% CI)**
Survival to Discharge	23.2%	28.9%	0.74 (0.71 to 0.78)
Acute Resuscitation Survival	60.3%	67.3%	0.74 (0.70 to 0.77)
Favorable Neurological Survival[a]	16.6%	21.1%	0.75 (0.70 to 0.79)

[a]Excluded 1,690 patients missing CPC score at discharge
